# Supplementary material for: Monocyte clusters suggestive of a chronic inflammatory phenotype are associated with reduced endothelial function in Veterans with respiratory symptoms
Source: PLoS One. 2026 Feb 10;21(2):e0338883. doi: 10.1371/journal.pone.0338883 (PMC12890113; doi:10.1371/journal.pone.0338883)

**S1 Fig. Extended gating strategy including broad population of B and T-cells.** (D) Within the lymphocyte population, B cells were defined based on their expression of CD19 and CD74. (E) T cells were identified using the pan T-cell marker CD3. Within the T cell population, subsets were further defined: T-helper cells (CD3+CD4+) and cytotoxic T cells (CD3+CD8+). Finally, activated lymphocytes were specifically gated by identifying cells expressing CD74 within the CD3+CD4+ and CD3+CD8+ populations.


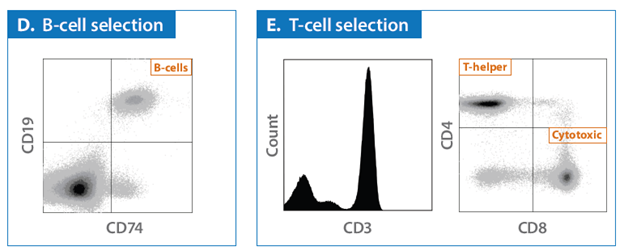

Supplement: S1 Fig — (DOCX) [file pone.0338883.s001.docx]
