# Supplementary material for: Monocyte clusters suggestive of a chronic inflammatory phenotype are associated with reduced endothelial function in Veterans with respiratory symptoms
Source: PLoS One. 2026 Feb 10;21(2):e0338883. doi: 10.1371/journal.pone.0338883 (PMC12890113; doi:10.1371/journal.pone.0338883)
Supplement: S1 Table — (DOCX) [file pone.0338883.s002.docx]

**S1 Table. mMRC (Modified Medical Research Council) Dyspnea Scale**

| 1 | Not troubled by breathlessness except during strenuous exercise |
| --- | --- |
| 2 | Short of breath when hurrying on the level or walking up a slight hill |
| 3 | Walking slower than most people on the level, stopping after one mile, or stopping after 15 minutes walking at own pace |
| 4 | Stopping for breath after walking about 100 yards or after a few minutes on level ground |
| 5 | Too breathless to leave the house, breathless when dressing or undressing |
