# Supplementary material for: Monocyte clusters suggestive of a chronic inflammatory phenotype are associated with reduced endothelial function in Veterans with respiratory symptoms
Source: PLoS One. 2026 Feb 10;21(2):e0338883. doi: 10.1371/journal.pone.0338883 (PMC12890113; doi:10.1371/journal.pone.0338883)
Supplement: S2 Table — (DOCX) [file pone.0338883.s003.docx]

**S2 Table. Selected CD Marker Overview.**

| **Marker** | **Cell Type** | **Description** |
| --- | --- | --- |
| **CD87** | Monocytes | Urokinase plasminogen activator receptor (uPAR), involved in cell signaling and migration. |
| **CD11b** | Monocytes | Integrin alpha M, involved in cell adhesion and migration. |
| **CD192** | Monocytes | CCR2, chemokine receptor involved in monocyte migration and recruitment. |
| **CD195** | Monocytes | CCR5, chemokine receptor involved in inflammation and immune response. |
| **HLADR** | Monocytes, B Cells, T Cells | MHC class II cell surface receptor, involved in antigen presentation. |
| **CD163** | Monocytes | Scavenger receptor, involved in anti-inflammatory responses and clearance of hemoglobin. |
| **CD3** | T Cells | Pan-T cell marker, present on all mature T cells. |
| **CD4** | T Cells | Marker for Helper T cells (Th) and some regulatory T cells (Tregs). |
| **CD8** | T Cells | Marker for Cytotoxic T cells |
| **CD19** | B Cells | Pan-B cell marker, on all B cells except plasma cells. |
| **CD74** | B Cells, T Cells, Antigen-presenting cells | Associated with MHC class II molecules, involved in antigen presentation. |
