# Supplementary material for: Monocyte clusters suggestive of a chronic inflammatory phenotype are associated with reduced endothelial function in Veterans with respiratory symptoms
Source: PLoS One. 2026 Feb 10;21(2):e0338883. doi: 10.1371/journal.pone.0338883 (PMC12890113; doi:10.1371/journal.pone.0338883)
Supplement: S3 Table — (DOCX) [file pone.0338883.s004.docx]

**S3 Table: Cell Subsets and Functions.**

| **Subset** | **Marker Combination** | **Potential Function** |
| --- | --- | --- |
| **Classical Monocytes** | CD14++CD16- | Phagocytosis, pro-inflammatory cytokine production, antigen presentation. |
| **Intermediate Monocytes** | CD14++CD16+ | Antigen presentation, moderate inflammatory responses, potential transitional state. |
| **Non-Classical Monocytes** | CD14+CD16++ | Patrolling endothelium, anti-inflammatory responses, tissue repair. |
| **Helper T Cells** | CD3+CD4+ | Help activate B cells, macrophages, and CD8+ Tcells; produce cytokines. |
| **Cytotoxic T Cells** | CD3+CD8+ | Kill virus-infected cells and tumor cells; produce cytotoxic granules. |
| **B Cells** | CD19+ | Produce antibodies, present antigens, and regulate immune responses. |
| **Activated B Cells** | CD19+CD74+ | Involved in antigen presentation and immune response modulation. |
| **Activated T Cells** | CD3+CD74+ | Involved in antigen presentation and possibly indicate an activated state of T cells. |
| **Helper T Cells (Activated)** | CD3+CD4+CD74+ | Activated helper T cells involved in antigen presentation and cytokine production. |
| **Cytotoxic T Cells (Activated)** | CD3+CD8+CD74+ | Activated cytotoxic T cells involved in antigen presentation and enhanced cytotoxic activity. |
