# Supplementary material for: Monocyte clusters suggestive of a chronic inflammatory phenotype are associated with reduced endothelial function in Veterans with respiratory symptoms
Source: PLoS One. 2026 Feb 10;21(2):e0338883. doi: 10.1371/journal.pone.0338883 (PMC12890113; doi:10.1371/journal.pone.0338883)
Supplement: S4 Table — (DOCX) [file pone.0338883.s005.docx]

**S4 Table. Pairwise comparisons of cluster 1 and cluster 2 for each marker, corrected for multiple comparisons.**

| Contrast | CD Marker | Estimate | SE | P-value |
| --- | --- | --- | --- | --- |
| Classical Monocytes  Cluster1 - Cluster2 | CD87  CD11b  CD192  CD195  HLADR  CD163 | 2.132  3.808  5.100  -0.164  0.854  1.006 | 0.395  1.030  1.045  0.094  0.808  0.414 | 0.000  0.000  0.000  0.086  0.294  0.017 |
| Intermediate Monocytes  Cluster1 - Cluster2 | CD87  CD11b  CD192  CD195  HLADR  CD163 | 1.447  1.819  0.993  -0.295  -1.006  0.633 | 0.287  0.615  0.535  0.087  5.575  0.320 | 0.000  0.004  0.067  0.001  0.857  0.051 |
| Non-classical Monocytes  Cluster1 - Cluster2 | CD87  CD11b  CD192  CD195  HLADR  CD163 | 0.850  0.453  -0.113  -1.904  -0.047  0.070 | 0.206  0.263  0.127  0.282  2.156  0.050 | 0.000  0.089  0.376  0.000  0.983  0.166 |
