# Supplementary material for: Monocyte clusters suggestive of a chronic inflammatory phenotype are associated with reduced endothelial function in Veterans with respiratory symptoms
Source: PLoS One. 2026 Feb 10;21(2):e0338883. doi: 10.1371/journal.pone.0338883 (PMC12890113; doi:10.1371/journal.pone.0338883)
Supplement: S5 Table — (DOCX) [file pone.0338883.s006.docx]

**S5 Table. Subcluster differences in CD surface marker expression.**

| **Characteristic** | **Overall**, N = 82*^1^* | **1A**, N = 13*^1^* | **1B**, N = 38*^1^* | **2A**, N = 20*^1^* | **2B**, N = 11*^1^* | **p-value***^2^* |
| --- | --- | --- | --- | --- | --- | --- |
| **Subset Frequencies (%)**  Classical  Intermediate  Non-Classical | 87(81.6, 90.0) 5 (3.04, 6.1)  8 (6.03, 11.9) | 90 (85.1, 93.3) 4 (2.42, 5.7)  6 (4.68, 9.1) | 87 (81.6, 90.4) 5 (2.89, 6.5)  8 (6.14, 11.5) | 85 (80.5, 87.8) 4 (3.4, 6.0)  10 (7.7, 13.4) | 87 (83.5, 88.9) 5 (3.13, 6.1)  8 (6.57, 10.8) | 0.2 0.7  0.073 |
| **CD87**  Classical  Intermediate  Non-Classical | 6 (5.4, 7.8)  6 (5.8, 7.6)  4 (3.6, 4.9) | 10 (8.99, 11.0)  9 (7.43, 9.9)  5 (4.40, 5.6) | 7 (6.0, 7.4)  7 (6.3, 7.5)  4 (3.9, 5.0) | 5 (4.3, 5.4)  5 (4.8, 6.0)  3 (2.7, 3.8) | 6 (5.63, 7.1)  7 (5.80, 7.4)  4 (3.68, 4.6) | <0.001  <0.001  <0.001 |
| **CD11b**  Classical  Intermediate  Non-Classical | 14(12.3, 15.8)  4(3.5, 5.2)  4(3.5, 5.2) | 21 (19.5, 24.8)  17 (14.6, 18.8)  5 (3.5, 5.3) | 15 (13.1, 16.6)  14 (12.7, 15.8)  4.7 (3.52, 5.3) | 11 (9.9, 11.7)  12 (11.3, 12.7)  4 (3.3, 4.4) | 16 (13.3, 18.8)  16 (14.0, 16.3)  3.9 (3.6, 5.1) | <0.001  <0.001  0.2 |
| **CD192**  Classical  Intermediate  Non-Classical | 19 (15.7, 23.0)  4 (3.2, 5.6)  0.8 (0.8, 0.8) | 23 (19.6, 27.0)  5 (3.7, 6.3)  0.8 (0.7, 0.8) | 21. (16.8, 25.1)  5 (3.6, 5.9)  0.8 (0.7, 0.8) | 15 (14.2, 17.6)  3.6 (2.9, 4.7)  0.8 (0.7, 0.9) | 17 (15.9, 19.8)  4 (3.1, 5.4)  0.8 (0.8, 1.2) | <0.001  0.2  0.017 |
| **CD195**  Classical  Intermediate  Non-Classical | 4 (3.7, 3.9)  4 (4.0, 4.5)  4(3.8, 5.1) | 4 (3.7, 3.9)  4 (3.9, 4.4)  4 (3.9, 4.5) | 4 (3.7, 3.9)  4 (4.0, 4.4)  4 (3.7, 4.3) | 4 (3.7, 3.9)  4 (4.3, 4.5)  5 (4.6, 5.4) | 4 (3.7, 4.1)  5 (4.3, 4.7)  7 (6.3, 9.1) | 0.2  0.005  <0.001 |
| **HLA-DR**  Classical  Intermediate  Non-Classical | 11 (8.5, 13.6)  80 (65.3, 101)  29 (22.1, 37.4) | 10 (8.2, 12.1)  68 (41.5, 79.8)  24 (20.4, 25.7) | 13 (10.3, 15.0)  82 (69.2, 110.4)  34 (22.52, 39.5) | 9 (8.1, 11.1)  76 (62.1, 91.1)  27 (21.1, 31.3) | 12 (11.1, 15.5)  100 (81, 109.4)  37(28.9, 38.1) | 0.007  0.012  0.005 |
| **CD163**  Classical  Intermediate  Non-Classical | 5 (3.9, 6.0)  4 (3.5, 5.1)  1 (1.1, 1.3) | 5 (4.8, 6.8)  4 (3.7, 6.5)  1 (1.0, 1.3) | 5 (4.0, 6.1)  5 (3.7, 5.4)  1 (1.1, 1.3) | 4 (3.0, 4.7)  4 (3.1, 4.2)  1 (1.1, 1.2) | 5 (4.6, 6.1)  5 (4.2, 5.4)  1 (1.0, 1.3) | <0.001  0.023  0.3 |
| *^1^*Median (IQR); *^2^*Kruskal-Wallis rank sum test | | | | | | |
|  | | | | | | |
