# Supplementary material for: Monocyte clusters suggestive of a chronic inflammatory phenotype are associated with reduced endothelial function in Veterans with respiratory symptoms
Source: PLoS One. 2026 Feb 10;21(2):e0338883. doi: 10.1371/journal.pone.0338883 (PMC12890113; doi:10.1371/journal.pone.0338883)
Supplement: S6 Table — (DOCX) [file pone.0338883.s007.docx]

**S6 Table. Pairwise comparisons of subcluster 1A and subcluster 1B for each marker, corrected for multiple comparisons.**

| Contrast | CD Marker | Estimate | SE | P-value |
| --- | --- | --- | --- | --- |
| Classical Monocytes  Subcluster 1A – Subcluster 1B | CD87  CD11b  CD192  CD195  HLADR  CD163 | 3.412 7.967 2.535 0.054 -2.529 1.312 | 0.375 1.024 1.458 0.129 1.072 0.553 | 0.000 0.000 0.517 1.000 0.125 0.121 |
| Intermediate Monocytes  Subcluster 1A – Subcluster 1B | CD87  CD11b  CD192  CD195  HLADR  CD163 | 1.744 2.855 0.838 0.040 -24.549 0.593 | 0.335 0.758 0.751 0.120 7.348 0.441 | 0.000 0.002 1.000 1.000 0.008 1.000 |
| Non-classical Monocytes  Subcluster 1A – Subcluster 1B | CD87  CD11b  CD192  CD195  HLADR  CD163 | 0.642 -0.029 -0.114 0.293 -9.059 -0.007 | 0.270 0.373 0.179 0.299 2.797 0.071 | 0.120 1.000 1.000 1.000 0.011 1.000 |
