# Supplementary material for: Monocyte clusters suggestive of a chronic inflammatory phenotype are associated with reduced endothelial function in Veterans with respiratory symptoms
Source: PLoS One. 2026 Feb 10;21(2):e0338883. doi: 10.1371/journal.pone.0338883 (PMC12890113; doi:10.1371/journal.pone.0338883)
Supplement: S7 Table — (DOCX) [file pone.0338883.s008.docx]

**S7 Table. Pairwise comparisons of subcluster 2A and subcluster 2B for each marker, corrected for multiple comparisons**.

| Contrast | CD Marker | Estimate | SE | P-value |
| --- | --- | --- | --- | --- |
| Classical Monocytes  Subcluster 2A – Subcluster 2B | CD87  CD11b  CD192  CD195  HLADR  CD163 | -1.757  -5.695  -1.435  -0.416  -3.274  -1.496 | 0.438  1.196  1.704  0.150  1.253  0.647 | 0.001  0.000  1.000  0.042  0.064  0.140 |
| Intermediate Monocytes  Subcluster 2A – Subcluster 2B | CD87  CD11b  CD192  CD195  HLADR  CD163 | -1.354  -3.137  -1.035  -0.329  -13.628  -1.024 | 0.391  0.885  0.878  0.141  8.584  0.515 | 0.005  0.004  1.000  0.132  0.699  0.302 |
| Non-classical Monocytes  Subcluster 2A – Subcluster 2B | CD87  CD11b  CD192  CD195  HLADR  CD163 | -0.922  -0.507  -0.296  -2.770  -8.100  -0.079 | 0.316  0.435  0.209  0.350  3.268  0.083 | 0.027  1.000  0.971  0.000  0.092  1.000 |
