# Supplementary material for: Monocyte clusters suggestive of a chronic inflammatory phenotype are associated with reduced endothelial function in Veterans with respiratory symptoms
Source: PLoS One. 2026 Feb 10;21(2):e0338883. doi: 10.1371/journal.pone.0338883 (PMC12890113; doi:10.1371/journal.pone.0338883)
Supplement: Attachment — Submitted filename: Response to Reviewers 2025-06-05.docx [file pone.0338883.s011.docx]

**Response to Academic Editor:**

Editor Kaminski,

Thank you for the opportunity to revise our work and resubmit for consideration. We have responded to your and the Reviewers’ comments below in bold.

1. Please ensure that your manuscript meets PLOS ONE's style requirements, including those for file naming.

**We have reviewed the style requirements to ensure our submission conforms, and have labeled our files accordingly.**

2. Please note that PLOS ONE has specific guidelines on code sharing for submissions in which author-generated code underpins the findings in the manuscript. In these cases, we expect all author-generated code to be made available without restrictions upon publication of the work.

**Our code for statistical analysis is now included in our submission.**

3. We note that the grant information you provided in the ‘Funding Information’ and ‘Financial Disclosure’ sections do not match.When you resubmit, please ensure that you provide the correct grant numbers for the awards you received for your study in the ‘Funding Information’ section.

**We added added the correct information in the Funding section.**

4. Please provide a complete Data Availability Statement in the submission form, ensuring you include all necessary access information or a reason for why you are unable to make your data freely accessible. If your research concerns only data provided within your submission, please write "All data are in the manuscript and/or supporting information files" as your Data Availability Statement.

**We have updated this info as below:**

**All data were collected at the VA and the signed subject consent forms did not make provision for making individual data records publicly available, even in de-identified form. However, the authors can provide the “metadata” – i.e. the numerical (aggregated data) results used to generate the figures. Requests for access can be sent to the authors.**

5. Please amend the manuscript submission data (via Edit Submission) to include author Carol Gardner, Changjiang (C.J.) Guo , Kathleen Black , Raymond Rancourt, Debra Laskin , Howard Kipen , Anays Sotolongo, Michael Falvo and Andrew Gow.

**All authors added.**

**Response to Reviewers:**

Reviewer #1: General comments: Although the manuscript includes some novel associations, such as the associations between lymphocyte clusters and flow mediated dilatation and oscillometry findings, the analysis of the other questionnaire-based data includes the analysis of many closely related questions (multiple categories of similar symptoms, for example). The also talk about drug development in the absence a specific disease is very premature.

**We appreciate the reviewer's observation about the number of closely related questions for symptoms and exposures. Our aim was to provide a comprehensive characterization of this cohort of veterans, who often present with a spectrum of health concerns, using standard instruments where possible. This approach was partly exploratory to understand the range of self-reported issues in the context of their monocyte profiles. It is an important finding that, despite these detailed assessments, the two immunologically distinct monocyte clusters generally did not differ significantly in most of these self-reported symptoms or exposures (as noted in Table 4). Our main conclusions are drawn from the significant associations observed between the monocyte clusters and the objective physiological measures (flow-mediated dilatation and oscillometry findings), rather than primarily from the questionnaire data.

We agree with the reviewer that discussing specific drug development is premature based on our findings. Our intention was not to suggest imminent drug therapies for a specific disease, but rather to propose that the identified chronic inflammatory monocyte phenotype and its association with physiological dysfunctions (like endothelial dysfunction and altered airway mechanics) might represent *potential areas for future research* into underlying mechanisms and, eventually, interventions. Our manuscript does state that 'more research is urgently needed to better understand the inflammatory pathways at play and to identify actionable targets'. We will carefully review the language in the Abstract, Discussion, and Conclusion to ensure it is appropriately speculative and emphasizes that our findings point towards the need for further mechanistic research before specific therapeutic strategies targeting monocytes can be considered for this population.**

Abstract: The phrase “Exposure to airborne hazards during deployment has been linked to persistent respiratory symptoms” is true, but the comment regarding vascular dysfunction is not common knowledge and is the basis of the current study. I also do not find the statement “Evidence suggests that circulating monocytes, key mediators of innate immune responses, contribute to these pathologic responses” in reference to respiratory symptoms is factual. I suggest rewriting the abstract to be more circumspect, focusing on the role of various environmental exposures on the immune system, and hypothesizing that such exposures may persist after exposure. This Veterans were studied years after exposure.

**We thank the reviewer for their detailed critique of the Abstract. We acknowledge the concerns raised and will revise the Abstract to be more circumspect, accurately reflect the nature of our findings as associations observed years after exposure, temper any suggestions of direct causality or immediate therapeutic translation, and better highlight the specific contribution of this research. We agree with the reviewer that the link between airborne hazard exposure and vascular dysfunction in veterans is an area of ongoing investigation rather than widely established common knowledge. Our study aimed to contribute to this emerging area. We will revise the opening of the Abstract to more accurately frame this. The reviewer also questions the direct factual link currently stated for monocytes contributing to respiratory symptoms. While our introduction builds a rationale for investigating monocytes due to their role in particulate matter-induced inflammation, which can affect respiratory health, we will rephrase this in the Abstract for greater precision.**

I also believe that finding association does not mean causality, and the statement “These results highlight the potential of monocyte-targeted interventions to mitigate chronic inflammation and downstream health effects in veterans with deployment-related exposures” is not warranted. We do not know the directionality of the associations they describe with inflammation, as many other cells and mediators are involved in the amplification of inflammation. The mention of therapy targeting monocyte activation is premature.

**We strongly agree that our cross-sectional study identifies associations and cannot imply causation. We also acknowledge that the mention of therapy targeting monocyte activation based solely on these findings is premature. We will significantly revise the sentence regarding interventions**

The last sentence is true of most author’s wishes “Further research is warranted, and should comment more on the significance of the research.

**We take the reviewer's point that the concluding sentence could more effectively highlight the significance of this research. We will revise it to be more impactful.**

These edits should be included in the discussion text

Introduction:
Line 54. What does “and its associated diversity” mean in regards to particulate matter.

Lines 56-58: “Mechanisms underlying post-deployment respiratory symptoms and conditions include activation of cell-signaling pathways in responsive cells leading to pro-inflammatory responses that occurs secondary to inhalation of PM (5,6). This also appears in the introduction, and needs editing, as noted in my previous comments.

Line 72: “and increased self-reported dyspnea”. Note that dyspnea is multifactorial, and may not always reflect vascular health.

Lines 73-75: “Understanding the inflammatory profiles of monocytes in this population could provide insights into both the pathogenesis of deployment-related respiratory disease and associated health outcomes, suggesting future approaches for targeted interventions.” Is premature, as the assessment on monocyte activation in the setting of inflammation may be the consequence of other inflammatory pathways, and needs additional linkage to disease

**We thank the reviewer for their thoughtful suggestions for text edits. All of the above have been appropriately amended in the text to reflect ‘softer’ language and further highlight the need for additional mechanistic research.**


Methods:
1. Were Veterans with cancer, receiving steroids/other immunomodulating drugs, or with chronic inflammatory conditions excluded?

- **Cancer: Yes, Veterans with a prior cancer diagnosis (excluding non-melanoma skin cancer) were excluded, as stated in our protocol and manuscript.**
- **Steroids/Immunomodulating Drugs: While not an explicit exclusion for all such drugs, participants with conditions often requiring these medications (e.g., certain active inflammatory diseases) would generally have been excluded under broader criteria outlined in our protocol, such as "history of pulmonary disease" or "cardiovascular disease." We will clarify in the manuscript that individuals with conditions necessitating systemic steroids or other immunomodulators that could significantly affect our outcomes were typically excluded based on their underlying diagnoses.**
- **Chronic Inflammatory Conditions: Yes, participants with specified chronic inflammatory conditions such as pulmonary disease, cardiovascular disease, and diabetes were excluded as detailed in the manuscript and protocol.**

**These criteria helped ensure a cohort suitable for our specific research questions. We will refine the manuscript to ensure these points are clear.**

2. Could past heavy smoking, in terms of having chronic inflammation in the lungs, in the absence of clinical disease, affect monocyte immunology? How was this addressed in the analysis?

**We thank the reviewer for highlighting the importance of considering past smoking. This was a large oversight on our end, and the updated analysis strengthened our results. In response to this comment, we have undertaken the following:**

1. **Re-Defined Smoking Variables: After reviewing available data, we have re-defined smoking status into the follow categories: Never Smoker: Individuals who have not smoked 100 cigarettes in their lifetime, or who don't know if they have, OR individuals who smoked 100+ cigarettes but never smoked regularly. Ever Smoker: Individuals who smoked 100+ cigarettes, smoked regularly, and have now quit. Note this is a more stringent definition of ever smoker then was previously used in our tables (prior calculations were based solely on ‘yes/no’ response to ‘have you ever smoked 100 cigarettes in your lifetime’). We believe this new categorization is more commonly used and offers better interpretation of the data. We also calculated 'Pack Years' for all former smokers (Number of cigarettes smoked per day / 20) * Number of years smoked).**
2. **Compared Smoking Variables Between Clusters: We statistically compared both 'Smoking Status' and 'Pack Years' (among former smokers) between Cluster 1 and Cluster 2. We found no significant differences in the distribution of these smoking variables between the two main clusters (these results will be added to Table 3 and mentioned in the text).**
3. **Updated Descriptive Statistics for Primary Outcomes: For our physiological outcomes (PFTs, FOT, and FMD/NMD – Tables 5-7), we now report the unadjusted Mean (SD) for each cluster and overall, instead of Median (IQR), to align with the subsequent modeling approach.**
4. **Re-analyzed Primary Outcomes with Adjusted Linear Models:**
   - **We fitted new linear models to assess the effect of Cluster on each outcome, including Smoking Status (Never vs. Ever) as a covariate to control for its potential confounding effect.**
   - **The p-values for the difference between Cluster 1 and Cluster 2 for these outcomes are now derived from these adjusted linear models, using the emmeans package for pairwise comparisons.**
   - **The corresponding effect sizes for the Cluster effect are also calculated from these adjusted models.**
5. **Updated text (methods), tables, and table legends.**

3. Did all subjects have land-based deployments?

**Land-based deployment was not part of our inclusion criteria, and we did not specifically ask this question. However, more than 82% of our sample endorsed being deployed to a base that had a burn pit and approximately 70% endorsed participating in convoy operations while deployed. These responses would suggest that the majority of our participants had land-based deployments.**

3. More information regarding spirometry procedures should be included. What predicted values were used for oscillometry?

**Regarding spirometry procedures: Our manuscript details that spirometry was performed pre- and post-bronchodilator using commercially available equipment (Cosmed, Quark PFT/Q-Box/i2M) by trained technicians. As stated, all testing followed the comprehensive guidelines published by the American Thoracic Society/European Respiratory Society (Miller et al., 2005;), which include detailed criteria for maneuver acceptability, repeatability, and quality control. Predicted values for FEV1, FVC, and FEV1/FVC were calculated using the Global Lung Function Initiative (GLI-2012) equations. We believe these details, in conjunction with the cited international guidelines, provide a thorough account of our spirometry methodology.

Regarding predicted values for oscillometry (FOT): We appreciate the reviewer highlighting the need to specify the source for our oscillometry predicted values. We used published reference equations to calculate predicted values for oscillometry parameters (R5, R20, X5, AX). Specifically, the coefficients and the form of the prediction equations:**

- **For resistance (e.g., predicted resistance = exp(a + b * height + c * age + d * weight))**
- **And for reactance (e.g., predicted reactance = 4 - exp(a + b * height + c * age + d * weight) for relevant frequencies) were taken from Oostveen E, et al. *Eur Respir J*. 2013;42(6):1513-1523. We implemented these equations using custom R scripts to derive the predicted values based on subject sex, height, age, and weight for the relevant frequencies. We have now added the precise citation for these reference equations to the Methods section of the manuscript.**

4. Suggest including detail on the MMRC questions in a supplement.

**We agree that providing its full wording can be beneficial for clarity. We have now included the complete mMRC Dyspnea Scale in the supplementary material (as Supplemental Table S1)**

5. More information regarding respiratory oscillometry (a more current description of what they did) is needed. What device was used?

**We have now revised the methods to provide a more detailed description of oscillometry, which is pasted below:**

***“Subjects also completed respiratory oscillometry (FOT), a non-invasive measure of respiratory mechanics assessed by the impedance of the respiratory system during tidal breathing. FOT was performed using optimized pseudorandom noise and a piezoresistive pressure transducer (Cosmed Quark i2M) while in a seated position, donning a noseclip, and providing manual cheek support in accordance with recommendations. A grading strategy was implemented to ensure data quality, prioritizing a coefficient of variation ≤ 10% between at least 3 trials. Predicted values for oscillometry parameters (R5, R20, X5, AX) were calculated using published reference equations.”***

6. More information on the protocols to collect and store monocytes are needed. How were batch effects handled in the 84 subjects?

**We thank the reviewer for this important question regarding our laboratory protocols and the handling of potential batch effects. Critically, all peripheral blood samples were processed, and monocyte immunophenotyping by flow cytometry was performed on fresh cells, on the same day of sample collection, for every participant over the study period. This continuous, fresh-sample workflow for each of the 84 subjects inherently avoids the creation of discrete experimental batches (e.g., those arising from batched cryopreservation, storage, and later analysis) that often necessitate computational batch effect adjustments.**

**To ensure data quality and consistency across the study's duration, standardized operating procedures were followed for all sample handling, staining, and flow cytometry acquisition. This included regular instrument calibration and performance checks, such as the daily us of single-stained controls to ensure consistent compensation setup and gating strategies. Given this same-day fresh analysis protocol for each individual sample and our efforts to maintain procedural consistency, specific post-hoc computational batch effect adjustments were not applied to the dataset.**

7. Shouldn’t time from deployment be calculated from time since last deployment, not the first one?

**We agree that 'time since last deployment' is an important metric, as it reflects the interval since the cessation of the most recent deployment-related exposures.**

**We would like to clarify that our study does indeed calculate and present both 'time since initial deployment' and 'time since last deployment.' These are reported in the overall sample characteristics (Table 1) and also compared between the two monocyte clusters (Table 3).**

**In our analysis comparing the demographic and deployment history characteristics between the two identified monocyte clusters (Table 3), we found that 'time since initial deployment' was significantly different between Cluster 1 (mean 13.7 ± 4.05 years) and Cluster 2 (mean 15.3 ± 3.29 years; p = 0.039). In contrast, 'time since last deployment' did not show a statistically significant difference between the clusters (Cluster 1: 11.1 ± 4.31 years; Cluster 2: 12.3 ± 3.91 years; p = 0.14). We highlighted 'time since initial deployment' in the text because it was the temporal metric that differed between our defined monocyte phenotype clusters.**

8. The exposure questions require some reference, as is not clear what burn pit duties means?

**We used items from the DARE (Deployment Airborne Respiratory Exposures) questionnaire developed by the US Army Public Health Command (reference). We have now revised the table to provide some specific examples of burn pit duties. It now reads as follows:**

***“Duties associated with the burn pit (e.g., trash burning, hauling trash, providing security at burn pit, trash sorting)”***

Results: Some of the groups/cells compared are tiny (like the race categories?) Why assess race? There were too many comparisons, many assessing similar outcomes (such as multiple dyspnea categories, multiple deployment related categories). Time since initial deployment was “significant” …. but how does one interpret this, as all categories of mean years since first deployment are quite similar?

**We acknowledge the reviewer's observation that some racial and ethnic categories in our cohort have small sample sizes, as detailed in Table 1 and Table 3. This indeed limits the statistical power for making meaningful comparisons or drawing conclusions about specific smaller racial/ethnic groups within our study.**

**Race and ethnicity were assessed and reported for several standard reasons: Cohort Characterization: To provide a comprehensive demographic description of our study population, which is standard practice in clinical research and important for assessing potential generalizability. Assessment of Distribution Between Clusters: We examined the distribution of race (and other demographics) between the two identified monocyte clusters to ensure that the clusters were not unduly confounded by demographic differences. As shown in Table 3, the overall racial distribution did not significantly differ between Cluster 1 and Cluster 2 (p=0.6). Context for Health Disparities: While not the primary focus of this immunological study, race and ethnicity can be associated with differential health outcomes or exposure risks in broader populations, making it relevant contextual information.**

**We did not perform sub-analyses based on the smaller racial categories due to limited statistical power. Our reporting focuses on the overall distribution and the comparison between the main study clusters.

The reviewer raises a valid point regarding the number of comparisons for self-reported symptoms and exposures (Table 4). Our study aimed to provide a comprehensive characterization of this Veteran cohort, many of whom experience a range of deployment-related health concerns. This involved using several questions from standardized assessments, such as the mMRC Dyspnea Scale and questions mirroring the VA’s Airborne Hazards and Open Burn Pit Registry self-assessment questionnaire, to capture various facets of their respiratory symptoms, functional limitations, and deployment exposures.**

**This approach was partly exploratory, intending to understand the breadth of self-reported issues within our immunologically defined clusters. Importantly, as stated in the results despite these multiple assessments, our clusters generally did not differ significantly in most self-reported respiratory symptoms or functional limitations (Table 4). This lack of strong association with self-reported symptoms, in contrast to the observed differences in physiological measures (FMD/NMD ratio and R5), is an important finding in itself. We interpret p-values for these descriptive comparisons with a standard alpha of 0.05, focusing on the overall pattern of findings rather than isolated significances among these secondary descriptive variables

The reviewer correctly notes that the absolute mean difference in 'time since initial deployment' between Cluster 1 (13.7 years) and Cluster 2 (15.3 years) is modest, at approximately 1.6 years. The statistical significance (p=0.039) indicates that this observed difference, while not large in absolute terms, is unlikely to have occurred by chance given our sample size and the variability within the groups. We reported this finding as it was the only demographic or deployment history characteristic (among those tested in Table 3) that showed a statistically significant difference between the two otherwise demographically similar monocyte clusters.**

**One potential interpretation, although speculative, is that a slightly longer period since the *initiation* of deployment-related exposures might contribute to the development or persistence of the chronic inflammatory monocyte phenotype observed in Cluster 2. Cluster 2 was characterized by higher expression of CD195 and a greater frequency of non-classical monocytes, suggestive of a chronic inflammatory state, and was associated with impaired endothelial function and increased airway resistance. While the difference is small, it may hint at long-term immunological sequelae. We acknowledge the modest nature of this difference and have aimed not to overstate its clinical importance but to report it as an observed statistical distinction between the clusters**

Discussion:
Lines 381-388: Given the heterogeneity of diagnosis in this population, I am concerned about making broad statements regarding the etiology of disease in this population, particularly with a small sample size. Even though they have no control group, there were participants without symptoms. What will the treatment trial focus on? There is no comment about the lack of association with symptoms or exposure…yet they talk of treatment?

**We appreciate the reviewer's caution regarding making broad statements about disease etiology, particularly given the heterogeneity of symptoms in veterans, our sample size, and the absence of a non-exposed/asymptomatic control group. We have acknowledged the lack of a control group as a key limitation in our Discussion section, noting that our findings are based on internal comparisons between the immunologically defined clusters.**

**Our study aimed to explore potential links between monocyte phenotypes and physiological changes in this specific cohort of deployed veterans with respiratory symptoms. While we 'suggest a link between chronic monocyte activation and microvascular and airway dysfunction' and discuss monocyte profiles 'potentially driving chronic inflammation', we strived to frame these as hypotheses requiring further validation. We believe our discussion is appropriately cautious and reflects the exploratory nature of these findings.

The reviewer correctly points out that our cohort includes individuals with a spectrum of symptom severity, with some participants not reporting significant dyspnea in daily living (e.g., approximately 72% had an mMRC score of 0 or 1). Our monocyte clusters were defined based on objective immunophenotypic markers, not pre-defined by symptom levels. A key finding is that these biologically distinct clusters were associated with significant differences in *objective physiological measures* - namely, endothelial-dependent vasodilation (FMD/NMD ratio) and airway resistance (R5) - irrespective of the self-reported symptom scores for individuals within those clusters at this cross-sectional time point. This suggests that the identified monocyte phenotypes may be linked to underlying, potentially subclinical, pathophysiological processes.

We agree with the critical point about the basis for discussing treatment. To clarify, our manuscript states that the two distinct monocyte clusters did not differ significantly in self-reported respiratory symptoms or deployment exposures. This is an important observation. However, the rationale for discussing the 'potential for monocyte-targeted therapies' stems from the significant association found between these immunologically defined monocyte clusters and objective physiological dysfunctions. These physiological impairments, even small, are relevant pathological findings that can contribute to long-term health issues.**

**Therefore, a potential future treatment trial might focus on:**

- **Target Population: Veterans who exhibit the 'Cluster 2-like' chronic inflammatory monocyte phenotype and/or the associated objective physiological dysfunctions (impaired FMD, elevated R5), rather than being selected solely on current self-reported symptom scores.**
- **Therapeutic Goal: To modulate monocyte activation state (i.e., shift away from the Cluster 2 phenotype) with the aim of improving these underlying physiological abnormalities (endothelial function, airway mechanics) and mitigating chronic inflammation.**
- **Endpoints: Improvements in these objective physiological measures, changes in monocyte activation markers, and potentially, over a longer term, an impact on symptom development, progression, or quality of life.**

**Our discussion of treatment emphasizes that 'more research is urgently needed to better understand the inflammatory pathways at play and to identify actionable targets,' underscoring the preliminary nature of this concept.**

Reviewer #2: Dear Author,

Your manuscript addresses a significant and often overlooked intersection between immunology, vascular biology, and military occupational health. The findings are innovative, and the methodology is sound. Your approach to using non-biased clustering of monocyte markers provides new perspectives on the chronic inflammatory mechanisms in post-deployment syndromes.

There are a few areas where further clarification or elaboration would enhance the manuscript:

• Control Group: The absence of a non-exposed or healthy control population is noted. Please consider discussing how this limitation affects the interpretation of your findings, and how future studies might address this, perhaps by including non-deployed veterans or matched civilian populations.

**We thank the reviewer for their positive assessment and insightful comments. Regarding the control group, we agree this is an important consideration. As noted in our manuscript's Limitations section, the absence of a non-exposed or healthy control group means our findings on monocyte phenotypes are relative to internal variations within our cohort of deployed veterans with respiratory symptoms. This affects interpretation in that we cannot determine if, for example, Cluster 1 represents a 'normal' healthy monocyte profile or simply a less altered state compared to Cluster 2. It also means we cannot establish absolute deviations from a non-deployed, asymptomatic baseline.**

**For future studies, we concur with the reviewer's suggestions. Incorporating control groups such as non-deployed veterans without respiratory symptoms, or appropriately matched civilian populations, would be invaluable. Ideally, longitudinal studies that include pre-deployment and multiple post-deployment assessments could also help establish individual baselines and track changes over time in response to exposures, though these are logistically challenging for historical exposures.**

• Sample Size in Functional Measures: The subset of participants who completed the FMD/NMD testing (n = 48) is relatively small. It would be helpful to clarify whether these individuals are representative of the larger cohort, and to note any demographic or clinical differences between those who did and did not complete these measures.

**We acknowledge the reviewer's point about the smaller sample size (n=48) for the flow-mediated dilation (FMD) and nitroglycerin-mediated dilation (NMD) procedures. To address whether this subset is representative of our larger cohort of 82 participants, we performed a comparative analysis. We** **compared key demographic characteristics (e.g., age, BMI, sex, smoking status) and self-reported deployment factors between participants who completed FMD/NMD testing and those who did not.**

**Our comparison revealed no statistically significant differences in age (p=0.4), BMI (p=0.9), sex distribution (p=0.9), race (p =0.4), smoking status (p=0.8), pack years (p = 0.6), mMRC (p = 0.3), total deployment time and time since both first and last deployments (all P >0.3) between** **those who underwent FMD/NMD testing and those who did not. This suggests that the subgroup for whom vascular function data are available is largely representative of the overall study cohort in these aspects. We have added this detail to the results section.**

• Temporal Disconnect: The immune data were collected long after deployment. While this is valuable in reflecting long-term effects, it also raises questions about immune memory versus ongoing inflammation. Could longitudinal immune profiling or additional biomarkers help distinguish between these?

**This is an excellent point. Our study, being cross-sectional with immune data collected years post-deployment, indeed captures long-term immunophenotypes but cannot definitively distinguish between stable, long-established 'immune memory' (perhaps in the sense of altered myeloid progenitor programming) versus more dynamically ongoing inflammatory processes reflected in circulating monocyte profiles. Given the relatively short lifespan of circulating monocytes, their surface marker expression at a single time point likely reflects a current or recent state of activation and differentiation, which could be indicative of an ongoing response or a persistently altered immune 'set-point.'**

**We agree with the reviewer that longitudinal immune profiling would be invaluable to track the dynamics of these monocyte phenotypes over time and better understand their persistence or evolution. Furthermore, incorporating additional biomarkers-such as plasma levels of relevant cytokines and chemokines, markers of endothelial activation, or even epigenetic markers within monocyte populations-could provide a more comprehensive picture and potentially help differentiate between stable alterations and active, ongoing inflammation. We have expanded our discussion of limitations to more explicitly address this point and the utility of future longitudinal designs with broader biomarker arrays.**

• Data Availability: Ensuring that the individual-level data used for PCA and clustering are placed in a publicly accessible repository or provided in supplementary files would greatly facilitate reproducibility.

**Unfortunately, we are not allowed to share individual-level data per our protocol. We have updated the data availability statement accordingly. However, we can make our code available.**

**Data Availability Statement:**

**All data were collected at the VA and the signed subject consent forms did not make provision for making individual data records publicly available, even in de-identified form. However, the authors can provide the “metadata” – i.e. the numerical (aggregated data) results used to generate the figures. Requests for access can be sent to the authors.**

Your study is strong and innovative, making a notable contribution to the literature. However, it could benefit from a more detailed discussion of its limitations, particularly regarding the lack of control data and the variability in sample size for sub-analyses. Clarifying the data availability would further strengthen the manuscript's impact and scientific rigour. I look forward to reviewing the revised version.
